# A Comprehensive Evaluation of Clinicopathologic Characteristics, Molecular Features and Prognosis in Lung Adenocarcinoma with an Acinar Component

**DOI:** 10.3390/cancers17111825

**Published:** 2025-05-30

**Authors:** Hanie Abolfathi, Manal Kordahi, Victoria Saavedra Armero, Nathalie Gaudreault, Dominique K. Boudreau, Andréanne Gagné, Michèle Orain, Pierre Oliver Fiset, Patrice Desmeules, Fabien Claude Lamaze, Yohan Bossé, Philippe Joubert

**Affiliations:** 1 Institut Universitaire de Cardiologie et de Pneumologie de Québec, Laval University, Quebec, QC G1V 4G5, Canada; hanie.abolfathi.1@ulaval.ca (H.A.); manal.kordahi.1@ulaval.ca (M.K.); victoria.saavedra-armero@criucpq.ulaval.ca (V.S.A.); nathalie.gaudreault@criucpq.ulaval.ca (N.G.); dominique.boudreau.4@ulaval.ca (D.K.B.); andreanne.gagne.4@ulaval.ca (A.G.); michele.orain@criucpq.ulaval.ca (M.O.); fabien.lamaze@criucpq.ulaval.ca (F.C.L.); yohan.bosse@criucpq.ulaval.ca (Y.B.); 2 Department of Pathology, McGill University, Montreal, QC H3A 0G4, Canada; pierre.o.fiset@mcgill.ca

**Keywords:** lung adenocarcinoma, acinar, gene mutations, prognosis, histological pattern

## Abstract

Introduction: Lung adenocarcinoma (LUAD) is the leading cause of cancer-related mortality worldwide. Acinar is the most prevalent architectural pattern and is associated with an intermediate prognosis. Several studies have investigated the prognosis of acinar-predominant LUAD patients. Here, we aimed to move beyond the acinar-predominant classification and gain a more comprehensive understanding of how acinar minor components influence prognosis specifically when accompanying other histological patterns in LUAD. Methods: Patients were grouped by the proportion of acinar patterns in their tumors: acinar-predominant (AP), and acinar component (AC; non-acinar predominant LUAD with an acinar component of ≥5%). The clinicopathologic characteristics, recurrence-free survival (RFS), and a panel of well-characterized driver mutations, including *KRAS*, *EGFR*, *BRAF*, *MET*, and *PIK3CA*, were investigated in the two groups of patients. Results: Among 1263 LUAD patients, 716 (56.7%) were AP, and 547 (43.3%) were AC. In AP, the frequency of *EGFR* exon 19 deletions (*EGFR-Del 19*) was significantly higher than in AC (*p* = 0.014). AC demonstrated a worse RFS than AP in the unadjusted analysis (log-rank *p*: 0.006). In stage I, the difference in the RFS of AC in comparison to AP remained significant (*p* = 0.048). In the multivariable analysis, AC was significantly associated with a worse RFS in comparison to AP (hazard ratio [HR] AC vs. AP: 1.240, 95% CI: 1.103–1.312, *p*: 0.04), even after adjusting for other histological patterns, the mutational status, and relevant clinicopathological features. The post-recurrence survival was significantly better in patients with an acinar component of ≥5% who received EGFR tyrosine kinase inhibitors (TKIs) compared to those who did not receive TKIs (*p* = 0.033). Conclusions: While the predominant pattern primarily dictates prognosis in LAUD, the presence of an acinar minor component alongside other high-grade patterns may further worsen outcomes. This underscores the necessity of considering the broader histological landscape rather than focusing solely on predominant patterns, as our findings show that minor acinar components can impact RFS alongside other histological patterns.

## Simple Summary

Lung adenocarcinoma (LUAD) is the most common type of lung cancer, and its prognosis often depends on the tumor’s microscopic structure. Acinar-predominant, the most frequent histological pattern, is associated with an intermediate prognosis. However, it remains unclear how minor acinar components influence patient outcomes. In this study, we examined over 1200 LUAD cases to compare patients with acinar-predominant tumors to those with tumors containing a minor acinar component. We analyzed the clinical characteristics, common driver mutations, and recurrence-free survival. We also evaluated the effect of EGFR tyrosine kinase inhibitors (TKIs) on post-recurrence survival in EGFR-mutated LUAD patients harboring an acinar component. Our results show that even small acinar components can worsen outcomes when combined with more aggressive patterns. This research suggests that looking beyond the predominant histological pattern and integrating molecular information may improve prognostic assessments and help guide personalized treatment decisions for patients with LUAD.

## 1. Introduction

Lung cancer is the most common cause of cancer-related death worldwide, among both men and women [1]. The most common histological type of lung cancer is LUAD, accounting for 40% of all lung cancer cases [2]. In 2011, a new classification based on six major architectural patterns was introduced for invasive and non-mucinous-LUAD (NM-LUAD), including lepidic, acinar, papillary, micropapillary, solid, and complex glandular patterns (CGPs; cribriform and fused gland) [3,4]. Each predominant pattern is associated with a distinct grade and prognosis (grade 1: lepidic-predominant; grade 2: acinar- or papillary-predominant; grade 3: solid and micropapillary-predominant). The International Association for the Study of Lung Cancer (IASLC) pathology committee updated this classification in 2021 to incorporate CGPs as a distinct category and redefined grade 3 tumors. According to the revised classification, grade 3 tumors must comprise at least 20% of high-grade patterns, such as solid, micropapillary, or CGPs [2,5]. The prognostic impact of the acinar pattern ranges widely, and identifying these CGPs may decrease the heterogeneity in the prognosis of acinar-predominant LUAD [6,7,8,9,10].

The acinar pattern is the most prevalent architectural pattern and consists of round-to-oval-shaped malignant glands invading the fibrous stroma [3,8]. Some specific tumor features, such as spread through air spaces (STAS) and lymphovascular invasion (LVI), are associated with acinar-predominant LUAD [11,12,13,14]. Molecularly, *EGFR* mutations are more frequently observed in acinar-predominant, whereas *KRAS* mutations are less common compared to their prevalence in solid and micropapillary patterns [15,16,17].

Studies suggest that the vast majority of LUAD cases (~80–90%) contain at least 5% of the acinar pattern, and acinar-predominant LUAD accounts for approximately 40–50% of all LUAD cases [18]. Patients with acinar-predominant tumors have an intermediate prognosis, better than micropapillary- or solid-predominant tumors, but worse than lepidic-predominant [14,19,20,21]. Earlier research has examined the prognostic value of LUAD with an acinar pattern in comparison to those without this pattern or acinar pr-dominant LUAD compared to other histologic subtypes.

While prior research has focused on acinar-predominant tumors, the prognostic significance of acinar components in non-acinar-predominant LUAD, especially alongside other histological patterns, remains unclear. Since minor histologic components can contribute to tumor behavior, a more comprehensive approach that considers both predominant and accompanying acinar patterns is essential for refining prognostic assessments.

This study leverages the updated IASLC grading system to refine the classification of acinar components in LUAD. To gain deeper insight into their prognostic importance, we investigated the clinicopathologic characteristics, molecular profiles, and outcomes of LUAD cases harboring an acinar component of at least 5%. Patients were categorized into two groups: acinar-predominant (AP), and acinar component (AC; non-acinar predominant LUAD with an acinar component of ≥5%).

Specifically, we first compared the clinicopathologic features and the frequency of common driver mutations between the two groups. We then conducted survival analyses focusing on recurrence-free survival (RFS) including stratified and multivariable models. Finally, we evaluated post-recurrence survival in EGFR-mutated LUAD patients with an acinar component of ≥5%, according to the administration of *EGFR* tyrosine kinase inhibitors (TKIs). This comprehensive approach allowed us to assess whether the presence of a minor acinar component influences prognosis when accompanying other histological patterns.

## 2. Materials and Methods

### 2.1. Study Population

A cohort of 1263 consecutive LUAD patients who underwent lung surgical resection between March 2006 and February 2021 was collected at the Institut universitaire de cardiologie et de pneumologie de Québec-Université Laval (IUCPQ-UL). The inclusion criteria were as follows: (1) diagnosis of LUAD; (2) complete surgical resection with negative margins (R0), and (3) the availability of hematoxylin and eosin (H&E) slides and tissue for histology and molecular characterization. The exclusion criteria were as follows: (1) patients who received neoadjuvant treatment, (2) those with multifocal or synchronous tumors, (3) cases of combined carcinomas, and (4) the cases with <5% acinar pattern. This project was approved by the IUCPQ-UL ethics committee (Number: MP-10-2022-3752, 22156).

### 2.2. Clinicopathological Data

Data on the clinical and pathological characteristics of the patients were collected. The following data were retrieved from the clinical chart: patient age, sex, smoking status, tumor location, type of surgical resection, and adjuvant therapy. RFS was defined as the time from surgery to the first recurrence or the last follow-up.

### 2.3. Histological Evaluation

The H&E slides from each tumor were retrieved and reviewed by thoracic pathologists (PJ, AG, PD, POF, MK). The grading followed the 2021 IASLC system for LUAD [2]. Patients were grouped by the proportion of acinar patterns in their tumors: acinar-predominant (AP), and acinar component (AC; non-acinar predominant LUAD with an acinar component of ≥5%). The following features were recorded: the architectural patterns, lymphovascular invasion (LVI), visceral pleural invasion (VPI), spread through air spaces (STAS), tumor size, and TNM stage (based on the 9th edition AJCC cancer staging) [22]. LUAD classification was based on the sum of six predominant patterns (acinar, papillary, solid, lepidic, micropapillary, and CGPs), totaling 100%.

### 2.4. Mutational Analysis

For the mutational analysis, DNA/RNA was extracted from either snap-frozen samples (*n* = 1109 for DNA and 1113 for RNA) or formalin-fixed paraffin-embedded (FFPE) samples (*n* = 154 for DNA and 150 for RNA). Nucleic acids were extracted and analyzed using the Oncomine™ Precision Assay GX Gene Panel (Thermo Fisher Scientific, Waltham, MA, USA), which includes 50 prevalent driver mutations and fusions in LUAD [23,24,25,26]. Next-generation sequencing (NGS) and alignment were conducted on the Ion Torrent Genexus platform according to the manufacturer’s recommendations. Each sample’s variant call report was reviewed by a pathologist (PJ or PD) for validation. To ensure the presence of tumors and assess tumor cellularity, a fraction of the samples was evaluated to determine the tumor content, with assessments primarily conducted using FFPE slides. All evaluated samples showed a tumor content of more than 10%.

### 2.5. Statistical Analysis and Visualization

To investigate the correlation of clinicopathological and molecular features with the acinar pattern components, the features were compared in AC and AP patients. The Mann–Whitney U test (Wilcoxon Rank Sum Test) was used to evaluate differences in continuous variables such as age and tumor size, while the Chi-square test or Fisher’s exact test was used for categorical variables to examine associations between the acinar pattern components and other clinicopathological features, as well as genetic variations. The RFS was analyzed using the Kaplan–Meier method, and group differences were evaluated with the log-rank test. To evaluate the prognostic value of the acinar pattern, the Cox proportional hazards regression model was used incorporating clinicopathological features, histological patterns, and molecular characteristics. All *p*-values were two-tailed, and a threshold of ≤0.05 was considered statistically significant. Statistical analyses were performed using the R statistical language (version 4.2.3, RStudio, Boston, MA, USA). Cox proportional hazards regression models, Kaplan–Meier analysis, and corresponding plots were generated using the R packages survival and survminer [27].

## 3. Results

### 3.1. Clinicopathologic Factors

**Figure 1:**
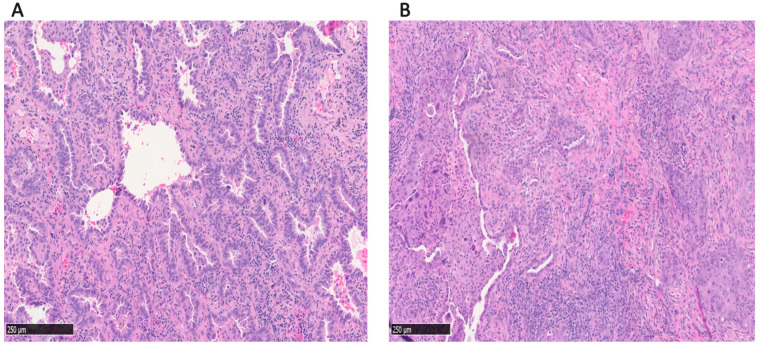
Representative images of (**A**) acinar-predominant and (**B**) acinar component; non-acinar-predominant LUAD with an acinar component of ≥5%.

The clinicopathologic characteristics of LUAD patients, both overall and based on acinar components, are presented in Table 1. A total of 1263 patients with LUAD were included in this study, comprising 716 AP and 547 AC cases. Representative images of AP and AC are shown in Figure 1. Of 547 AC patients, the predominant histologic subtype was lepidic in 136 patients, papillary in 50 patients, and ≥20% solid, micropapillary, or CGPs in 361 patients.

Among all, 785 (62.1%) were female and 478 (37.9%) were male, ranging in age from 26 to 88 years (median: 66 years). The distribution of tumor stages was as follows: 923 (73.1%) cases were stage I, 219 (17.3%) were stage II, and 121 (9.6%) were stage III.

Compared to AP, AC cases had a significantly lower proportion of stage I disease (69.5% vs. 75.9%) and a higher proportion of stage III disease (12.2% vs. 7.5%) (*p* = 0.009). The median tumor size was slightly larger in AC (2.5 cm) than in AP (2.3 cm, *p* = 0.0009).

Histopathologically, there were significant differences in the tumor grade distribution between AP and AC groups (*p* < 0.00001). Grade 3 tumors were significantly more common in AC than in AP (66.0% vs. 45.9%).

Tumor localization differed significantly between the groups (*p* = 0.045). AP tumors were more frequently located in the right upper lobe (41.2% vs. 34.5%), while AC tumors were more common in the right lower lobe (18.8% vs. 14.1%). These findings suggest potential differences in the tumor origin or spread patterns between histologic subtypes.

To further investigate whether the observed differences were specific to the acinar type or could be attributed to the presence of other histologic subtypes, we performed an additional comparative analysis including cases with acinar-predominant, papillary-predominant, or lepidic-predominant and tumors with ≥20% high-grade patterns (solid, micropapillary, or CGPs), together (Supplementary Table S1).

We found that the *EGFR-Del19* mutation, which was significantly different between AP and AC, was not different across all histologic patterns, suggesting that these differences may be intrinsic to the acinar type. Conversely, features like LVI, VPI, and STAS were not significantly different between AP and AC but showed differences among the subgroups of all histological patterns, suggesting that these may be driven by the presence of high-grade and not intrinsic to the acinar type.

### 3.2. Status of Common Driver Mutations

We evaluated the frequencies of the most common driver mutations, including *KRAS*, *EGFR*, *BRAF*, *MET*, and *PIK3CA*, by sub-mutations. Less frequent mutations, such as those in *ARAF*, *CTNNB1*, *ERBB2*, *MAP2K1*, *NRAS*, *GNAS*, and *FGFR2/3*, were grouped together as “Other”. Among the 1263 LUAD cases, 907 cases had a detected driver mutation, while 356 cases were classified as wild-type (WT) with no detected mutations. The most frequently observed driver mutations were *KRAS-G12C* (20.9%), *KRAS-G12V* (9.4%), *KRAS-G12D* (4.9%), *EGFR-L858R* (4.9%), and *EGFR-Del-19* (4.7%).

**Figure 2:**
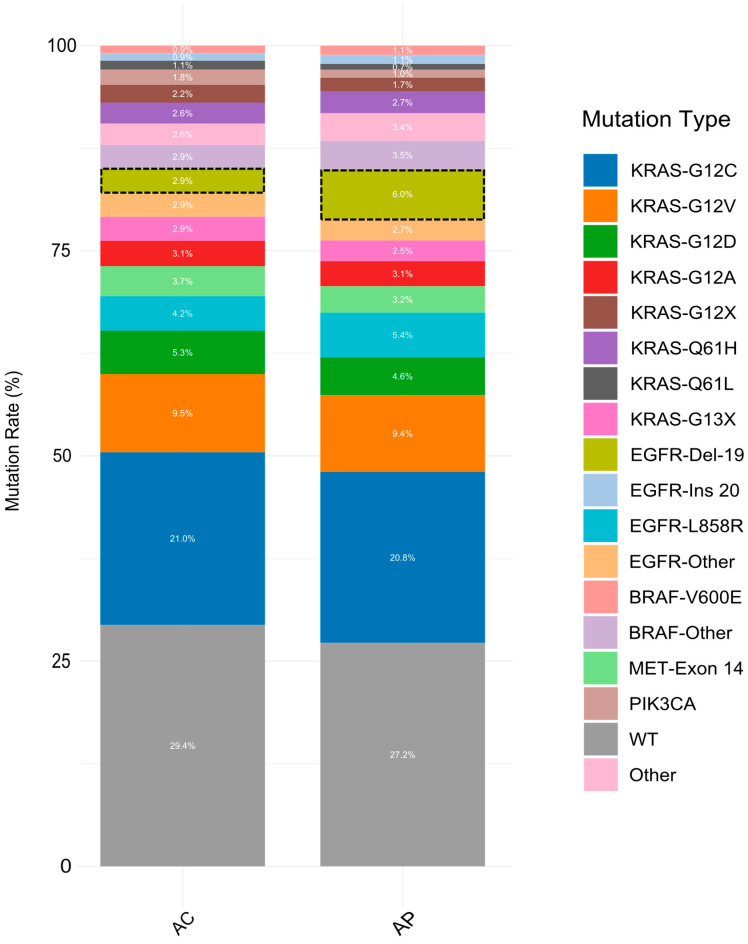
Summary of the most common driver mutations in LUAD patients in two groups of AP and AC. AP: acinar-predominant, AC: acinar component (non-acinar predominant LUAD with an acinar component of ≥5%), WT: wild-type, other refers to the less frequent mutations, such as those in *ARAF*, *CTNNB1*, *ERBB2*, *MAP2K1*, *NRAS*, *GNAS*, and *FGFR2/3*. Note: The dotted squares represent the statistically significant difference in the frequency of *EGFR-Del 19* mutations between the two groups of AP and AC (*p* = 0.014).

To investigate the correlation between molecular features and acinar pattern components, we compared the frequency of the most prevalent driver mutations between the two groups of AP and AC. Among the analyzed mutations, only the frequency of the *EGFR-Del-19* mutation demonstrated a statistically significant difference between the two groups (*p* = 0.014) (Figure 2, Table 1).

As *EGFR-Del-19* was the only mutation significantly correlated with the acinar histology among all evaluated mutations, we further assessed its relationship with the acinar pattern components through univariable and multivariable logistic regression analysis (Table 2). Since the AC group is heterogeneous and includes other predominant histological patterns, we used a multivariable model to adjust for these histological patterns, along with relevant clinical and pathological variables.

The results indicated that the presence of the *EGFR-Del-19* mutation remained significantly associated with the acinar pattern even after an adjustment (Table 2). Specifically, AP was associated with a significantly higher likelihood of the presence of *EGFR-Del-19* compared to AC in the multivariable model (OR = 1.951, 95% CI: 1.701–2.205, *p* = 0.03).

These findings highlight the molecular heterogeneity within LUAD and underscore the importance of considering minor components of the acinar type beyond the predominant classification. Since non-predominant acinar components may influence the mutational landscape, their presence should be assessed alongside other histological patterns to refine prognostic evaluations and potential therapeutic strategies. Incorporating AC provides a more comprehensive understanding, as AC was associated with a significantly lower likelihood of the presence of *EGFR-Del-19*, even after adjusting for other histological patterns and clinicopathological features.

### 3.3. Survival Analysis

For the survival analysis, our primary endpoint was RFS. In our cohort, 73% of patients were diagnosed at stage I. In early-stage LUAD, RFS is a more relevant measure of tumor aggressiveness and treatment efficacy. RFS provides valuable insights into disease recurrence patterns, which have direct implications for clinical decision-making, including adjuvant therapy strategies. By focusing on RFS, we aim to better assess the prognostic value of ACs in LUAD while minimizing external influences on survival outcomes.

**Figure 3:**
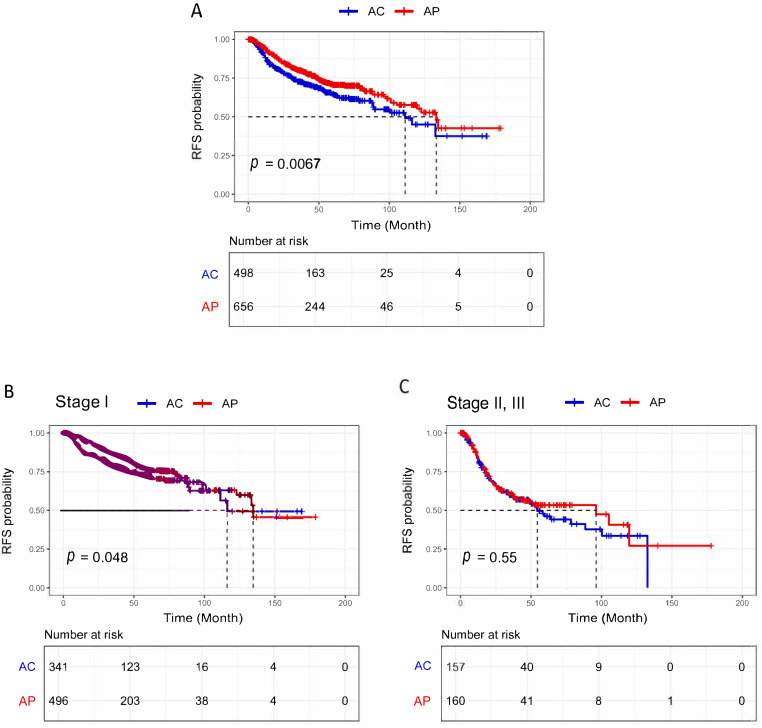
(**A**) RFS probability of all patients, and (**B**) RFS probability of stage I, and (**C**) Stage II, III. AP: acinar-predominant, AC: acinar component (non-acinar predominant LUAD with an acinar component of ≥5%), RFS: recurrence-free survival. Note: “*p*” represents the log-rank *p*-value, which was calculated through an unadjusted analysis (log-rank test). The dotted lines indicate the time (in months) at which the RFS probability reaches 0.5 for each group, representing the median RFS.

Among all, 1154 (91.3%) had at least one post-surgery visit to calculate the RFS. Survival outcomes were compared between 716 patients with AP and 547 patients with AC. The median follow-up time (95% CI) of these patients was 122.7 months (105-NA), and the recurrence rate was 27% (312 cases). The median RFS time (95% CI) was 133.3 (119.7-NA) and 111.2 (88.6-NA) for AP and AC patients, respectively. Among all, 163 (22.7%) AP and 149 (27.2%) AC patients had recurrent disease. In the unadjusted analysis (log-rank test), AC LUAD patients had a significantly worse RFS (*p* = 0.006) than AP patients (Figure 3A).

The survival analysis was followed by stratifying cases based on the TNM stage to stage I (Figure 3B) and stages II–III (Figure 3C). Compared to AP patients, AC patients had a significantly worse RFS in the stages I group (*p* = 0.048, HR: 0.744, 95% CI: 0.674–0.822) (Figure 3C).

We then focused on the univariable and multivariable analysis of RFS using the Cox regression hazards model (Table 3). In the univariate analysis, AC had a worse RFS than AP [HR AC vs. AP: 1.358, 95% CI: 1.188–1.541, *p*: 0.006]. An older age at diagnosis (*p* = 0.002), TNM stage (II–III vs. I, *p* = 2.76 × 10^−15^), higher tumor grade (3 vs. 1, 2, *p* = 3.38 × 10^−9^), the presence of LVI (*p* = 2 × 10^−8^), VPI (*p* = 0.0005), STAS (*p* = 0.003), and the presence of solid-predominant (*p* = 0.0001), micropapillary-predominant (*p* = 0.005), and CGP-predominant (0.005) were significantly associated with a worse RFS. In contrast, the presence of lepidic-predominant (*p* = 4.1 × 10^−6^) was associated with an improved RFS.

Of the 547 AC patients, the predominant histologic subtype was lepidic in 120 patients, papillary in 49 patients, and ≥20% solid, micropapillary, or CGPs in 329 patients. We performed a multivariable analysis adjusting for variables that showed a significant association with RFS in the univariable analysis. These included age, TNM stage, tumor grade, LVI, VPI, STAS, and the histological patterns of lepidic, micropapillary, solid, and CGPs. We selected AC as the reference group and compared it with AP.

In the multivariate analysis, acinar (HR AC vs. AP = 1.240, 95% CI: 1.103–1.312, *p* = 0.04), TNM stage (HR _II, III vs. I_ = 1.830, 95% CI: 1.533–2.118, *p* = 1.25 × 10^−6^), and older age (HR = 1.293, 95% CI: 1.199–1.321, *p* = 0.014) were the independent predictors of a worse RFS. In addition, the presence of micropapillary-predominant (HR = 1.296, 95% CI: 1.107–1.367, *p* = 0.043) was significantly associated with a worse RFS. However, solid-predominant (*p* = 0.951) and CGP-predominant (*p* = 0.303) did not show a significant impact on the RFS. This finding suggests that micropapillary, a high-grade pattern associated with a worse prognosis, can lead to a poorer RFS when accompanied by a minor acinar component.

**Figure 4:**
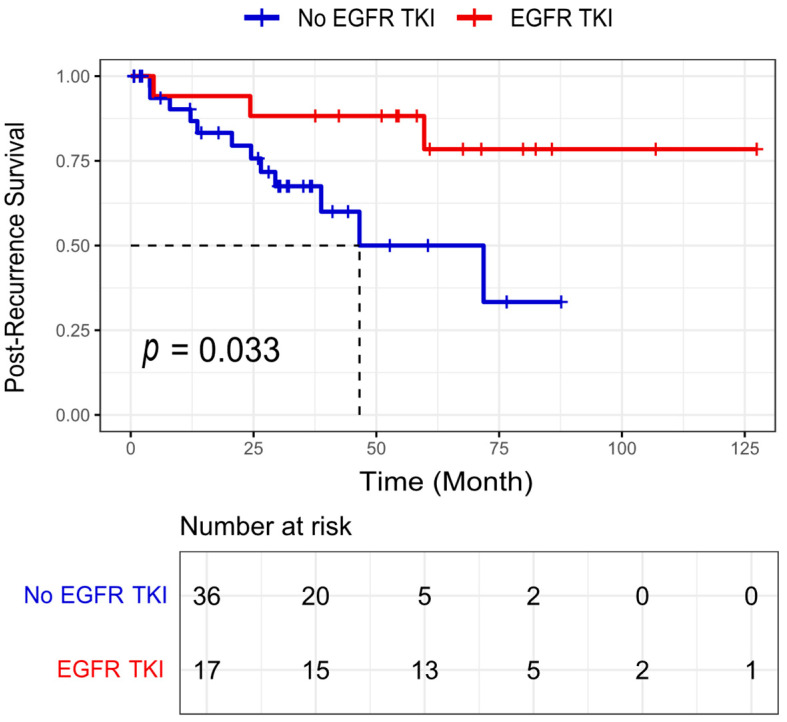
Post-recurrence survival of patients with EGFR-mutated lung adenocarcinoma harboring an acinar component of ≥5% according to the administration of EGFR tyrosine kinase inhibitors (TKI). Note: “*p*” represents the log-rank *p*-value, which was calculated via an unadjusted analysis (log-rank test). EGFR-TKI refers to cases that received EGFR-TKIs (gefitinib or erlotinib), while No EGFR-TKI refers to cases that did not receive these treatments. The dotted lines indicate the time (in months) at which the post-recurrence survival probability reaches 0.5 for each group, representing the median post-recurrence survival.

We further evaluated the effect of EGFR-TKIs on post-recurrence survival in EGFR-mutated LUAD patients harboring an acinar component of ≥5% (Figure 4). Among 53 patients who experienced recurrence during follow-up, 17 received EGFR-TKI therapy (gefitinib or erlotinib). An objective response was observed in 12 of the 17 patients (1 complete response and 11 partial responses), while the remaining 5 had stable disease at the first evaluation. The median post-recurrence survival was not reached (NA) in the EGFR-TKI group compared to 46.9 months in the non-TKI group. The post-recurrence survival was significantly better in patients who received EGFR-TKIs compared to those who did not (log-rank *p* = 0.033). These findings support the potential clinical benefit of EGFR-TKI therapy for recurrent EGFR-mutated LUAD with an acinar component and highlight the importance of molecular testing in guiding post-recurrence treatment decisions.

Our findings highlight the heterogeneity of the AC group, showing that prognosis is primarily dictated by the predominant pattern, but the minor components of acinar alongside high-grade patterns can worsen outcomes. While AC alone is not an independent prognostic factor, its presence alongside other predominant patterns contributes to a worse prognosis, reinforcing the importance of comprehensive histopathological evaluations in LUAD.

## 4. Discussion

Based on the IASLC/ATS/ERS classification system, LUAD resection specimens are classified based on the predominant histologic pattern, following a comprehensive histologic evaluation that involves subtyping in 5% increments of each architecture pattern [3,28]. These patterns include lepidic, papillary, acinar, solid, micropapillary, and CGPs. In 2021, the previous grading system of LUAD was updated by introducing CGPs and a new definition of grade 3 tumors to better prognosticate patient evolution [2]. The acinar pattern is the most common architectural pattern in LUAD and is associated with an intermediate prognosis. Previous studies have examined the prognostic value of acinar-predominant LUAD compared to other histologic subtypes [14,20].

Given the heterogeneity of LUAD, the prognosis is primarily dictated by the predominant histologic pattern. However, the minor acinar components coexist with other patterns in 40–50% of all LUAD cases, raising the question of whether their presence influences outcomes beyond the predominant classification. While prior research has focused on acinar-predominant tumors, little is known about the prognostic significance of AC in non-acinar-predominant LUAD. Since minor histologic components can contribute to tumor behavior, a more comprehensive approach that considers both predominant and accompanying patterns is essential for refining prognostic assessments.

As far as we know, this is the first study to compare AP with non-acinar-predominant LUAD containing an acinar component of at least 5%, focusing on the clinicopathologic characteristics, mutational spectrum, and RFS. This study can offer valuable insights for assessing the relapse risk after surgical resection.

The distribution of gene mutations and rearrangements in LUAD-predominant histologic subtypes is well-documented. Notably, *EGFR* mutations are significantly more prevalent in AP-LUAD [15,16,17,29,30,31]. In this study, we evaluated the frequency of the most common driver mutations, including *KRAS*, *EGFR*, *BRAF*, *MET*, and *PIK3CA*, by sub-mutations. we demonstrated that the frequency of the *EGFR-Del 19* mutation is higher in AP-LUAD than in AC. Specifically, AP was associated with a significantly higher likelihood of the presence of *EGFR-Del-19* compared to AC in the multivariable model with an adjustment for the other histological patterns and clinicopathological features.

Since non-predominant acinar components may influence the mutational landscape, their presence should be assessed alongside other histological patterns to refine prognostic evaluations and potential targeted therapies. Incorporating the AC provides a more comprehensive understanding, as AC was associated with a significantly lower likelihood of the presence of *EGFR-Del-19*, even after adjusting for other histological patterns and clinicopathological features.

AP-LUAD has been reported to exhibit specific clinicopathological characteristics [11,12,13,32]. Caso et al. found that among AP-LUAD tumors, VPI was independently associated with an increased risk of recurrence [11]. Kim et al. identified STAS as an independent prognostic biomarker for RFS in an AP-LUAD cohort [14]. In our study, the frequencies of STAS, LVI, and VPI did not differ significantly between AP and AC.

Previous studies have shown that patients with AP had better overall survival (OS) than those with solid, micropapillary, or CGP-predominant patterns [14,19,20] but worse OS than those with lepidic-predominant patterns [33]. Our specific focus was on the comparison between AP tumors and non-acinar-predominant tumors with an acinar component of ≥5%, adjusting for other histological patterns and clinicopathological features.

For the survival analysis, our primary endpoint was RFS rather than OS for several reasons. First, in early-stage LUAD, RFS is a more relevant measure of tumor aggressiveness and treatment efficacy, as OS can be influenced by non-cancer-related factors, such as comorbidities and unrelated causes of death. Second, RFS provides valuable insights into disease recurrence patterns, which have direct implications for clinical decision-making, including adjuvant therapy strategies. Third, RFS events occur earlier than OS events, reducing the impact of confounding factors, such as variations in post-recurrence treatment and prolonged follow-up durations. Notably, in our cohort, 73% of patients were diagnosed at stage I, further supporting the use of RFS as a more appropriate endpoint to evaluate early disease progression and recurrence risk. By focusing on RFS, we aim to better assess the prognostic value of acinar patterns in LUAD while minimizing external influences on survival outcomes.

We found that AP patients had significantly better RFS than AC patients. In our cohort, the AC group had a higher proportion of high-grade tumor patterns, including solid and micropapillary, which are associated with poorer prognosis. To address this imbalance, we performed a multivariable analysis adjusting for the mutational status, clinicopathological variables, and other histopathological patterns, including the high-grade patterns. We selected *KRAS*, *EGFR*, *BRAF*, and *MET* mutations for inclusion in the multivariable model because they are among the most commonly altered driver mutations in LUAD and are known to significantly influence tumor behavior, prognosis, and response to targeted therapies. Even after the adjustment, AC continued to show a worse RFS than AP. When survival analysis was stratified by stage, the difference in RFS between AC and AP remained significant in stage I and not in stage II, III. Additionally, the presence of micropapillary-predominant was associated with a worse RFS, suggesting that even minor acinar components can amplify the adverse prognostic impact of the micropapillary-predominant type, further worsening RFS.

Our findings highlight the heterogeneity of the AC group, demonstrating that while AC alone is not an independent prognostic factor, prognosis is primarily dictated by the predominant pattern. However, the presence of a minor acinar component alongside high-grade patterns can exacerbate poor outcomes.

Several clinicopathological features, including the TNM stage, lymph node metastasis, tumor size, and histological patterns, were shown to be associated with the response to targeted therapy in LUAD [31,34]. The AP was shown to have an intermediate response to adjuvant therapy, better than solid and micropapillary, but worse than lepidic [2,35]. In our study, we examined the effect of EGFR-TKIs on the post-recurrence survival of patients with EGFR-mutated LUAD harboring an acinar component of ≥5%. The post-recurrence survival was significantly better in patients who received EGFR-TKIs compared to those who did not receive TKIs.

Our results do not suggest that the acinar component alone serves as an independent prognostic factor, given its heterogeneity. Instead, when considered alongside other histological patterns, the presence of a minor acinar component can contribute to a worse prognosis. This reinforces the importance of evaluating minor acinar components within the comprehensive histopathological context to refine prognostic assessments and treatment strategies in LUAD.

Unlike previous studies that primarily focused on acinar-predominant LUAD or compared histologic subtypes without considering coexisting patterns, our methodology offers a more granular and comprehensive approach by isolating the prognostic role of acinar components in non-acinar-predominant tumors. A key strength of our study is the integration of a detailed histological assessment with advanced molecular and clinical analyses. We employed the Oncomine™ Precision Assay GX, a broad and clinically validated next-generation sequencing panel, to evaluate a wide spectrum of prevalent driver mutations in LUAD, including *EGFR*, *KRAS*, *BRAF*, *MET*, and *PIK3CA*. Importantly, we analyzed associations at the level of specific sub-mutations, such as *EGFR-Del19* and *KRAS-G12C*, allowing for deeper genotype–phenotype correlations than previous studies. Furthermore, we applied the 9th edition of the TNM staging system, recently proposed in 2024, which enhances the accuracy of the prognostic classification. By adjusting for coexisting high-grade histological patterns and incorporating post-recurrence treatment outcomes, our study provides a refined and clinically actionable stratification framework for LUAD patients that surpasses conventional predominant-pattern-based approaches.

Our study has limitations. The prevalence of EGFR-TKI therapy was low, with only 53 cases qualifying for an evaluation of these modalities. A larger prospective clinical trial would be valuable to validate our findings and further explore the impact of these therapies on the prognosis of acinar-predominant LUAD patients.

Pathology reviews are typically conducted by a single pathologist to minimize variability in interpretation. In this study, five pathologists were involved, which may have increased inter-observer variability, as differences between reviewers are well-documented in the literature. However, in cases of disagreement, the pathologists discussed their findings to reach a consensus, likely reducing the impact of this variability. Lastly, the genotypes and phenotypes of patients may be influenced by ethnicity. In this study, all cases were French-Canadian. Therefore, conducting similar studies with more diverse populations would be valuable to validate our results across different ethnic backgrounds.

## 5. Conclusions

In conclusion, our study highlights the importance of considering the minor components of the acinar pattern in the prognosis and management of LUAD patients. Although the AP LUAD is used in clinical analysis, its minor components may have an impact on the prognosis alongside other histological patterns. For stage I, AC was even more likely to recur than AP and should be distinguished from AP. Additionally, our results underscore the potential benefit of EGFR-TKI therapy for EGFR-mutated LUAD with acinar components, suggesting the importance of integrating molecular data with histopathological classification for therapeutic decision-making. Future studies should focus on validating these findings using multi-ethnic and prospective cohorts and exploring the biological mechanisms by which minor acinar components interact with high-grade patterns to influence tumor behavior. Further investigation into the predictive role of specific mutations in relation to histologic subtypes could also inform personalized therapeutic strategies.

## Figures and Tables

**Table 1:** Characteristics of LUAD patients, overall and based on acinar components.

Characteristics	Overall (*N* = 1263)	AP (*N* = 716)	AC (*N* = 547)	*p*-Value (AP vs. AC)
***Age***				
*Median*	*66*	*66*	*66*	0.34
*Min–Max*	*26–88*	*37–88*	*26–84*	
**Sex**				
Male	478 (37.9%)	255 (35.6%)	225 (41.1%)	**0.045**
Female	785 (62.1%)	461 (64.4%)	322 (58.9%)	
**Smoking status**				
Ever	1194 (94.5%)	675 (94.3%)	519 (94.9%)	0.637
Never	69 (5.5%)	41 (5.7%)	28 (5.1%)	
**N status**				
N0	497 (39.3%)	270 (37.7%)	227 (41.5%)	0.171
N1/2	766 (60.7%)	446 (62.3%)	320 (58.5%)	
**Stage**				**0.009**
I	923 (73.1%)	543 (75.9%)	380 (69.5%)	
II	219 (17.3%)	119 (16.6%)	100 (18.3%)	
III	121 (9.6%)	54 (7.5%)	67 (12.2%)	
***Tumor size (cm)***				
*Median*	*2.3*	*2.3*	*2.5*	**0.0009**
*Range*	*1–15*	*1–15*	*1–12.5*	
**Grade**				**<0.00001**
1	136 (10.8%)	0 (0.0%)	136 (24.9%)	
2	437 (34.6%)	387 (54.1%)	50 (9.1%)	
3	690 (54.6%)	329 (45.9%)	361 (66.0%)	
**Type of surgery**				0.961
Lobectomy	954 (75.6%)	539 (75.3%)	415 (75.9%)	
Segmentectomy	132 (10.5%)	75 (10.5%)	57 (10.4%)	
Other	177 (14.0%)	102 (14.3%)	75 (13.7%)	
**Tumor localization**				**0.045**
Left Upper Lobe	323 (25.6%)	186 (26.0%)	137 (25.1%)	
Left Lower Lobe	153 (12.1%)	84 (11.7%)	69 (12.6%)	
Right Upper Lobe	486 (38.5%)	295 (41.2%)	189 (34.5%)	
Right Lower Lobe	202 (16.0%)	101 (14.1%)	103 (18.8%)	
Other	99 (7.8%)	50 (7.0%)	49 (9.0%)	
**STAS**				
Yes	537 (42.5%)	312 (43.6%)	225 (41.1%)	0.384
No	726 (57.5%)	404 (56.4%)	322 (58.9%)	
**LVI**				
Yes	592 (46.9%)	329 (46.0%)	263 (48.1%)	0.452
No	571 (45.2%)	387 (54.0%)	284 (51.9%)	
**VPI**				
Yes	343 (27.2%)	194 (27.1%)	149 (27.2%)	0.954
No	920 (72.8%)	522 (72.9%)	398 (72.8%)	
**Mutational status**				
KRAS-G12C	264 (20.9%)	149 (20.8%)	115 (21.0%)	0.981
KRAS-G12V	119 (9.4%)	67 (9.4%)	52 (9.5%)	1.000
KRAS-G12D	62 (4.9%)	33 (4.6%)	29 (5.3%)	0.664
KRAS-G12A	39 (3.1%)	22 (3.1%)	17 (3.1%)	1.000
KRAS-G12X	24 (1.9%)	12 (1.7%)	12 (2.2%)	0.645
KRAS-G13X	34 (2.7%)	18 (2.5%)	16 (2.9%)	0.785
KRAS-Q61H	33 (2.6%)	19 (2.7%)	14 (2.6%)	1.000
KRAS-Q61L	11 (0.9%)	5 (0.7%)	6 (1.1%)	0.652
EGFR-Del-19	59 (4.7%)	43 (6.0%)	16 (2.9%)	**0.014**
EGFR-L858R	62 (4.9%)	39 (5.4%)	23 (4.2%)	0.378
EGFR-Ins 20	13 (1.0%)	8 (1.1%)	5 (0.9%)	0.941
EGFR-Other	35 (2.8%)	19 (2.7%)	16 (2.9%)	0.905
MET-Exon 14	43 (3.4%)	23 (3.2%)	20 (3.7%)	0.783
BRAF-V600E	13 (1.0%)	8 (1.1%)	5 (0.9%)	0.941
BRAF-Other	41 (3.2%)	25 (3.5%)	16 (2.9%)	0.687
PIK3CA	17 (1.3%)	7 (1.0%)	10 (1.8%)	0.292
Other	38 (3.0%)	24 (3.4%)	14 (2.6%)	0.515
WT	356 (28.2%)	195 (27.2%)	161 (29.4%)	0.425

AP: acinar-predominant, AC: acinar component (non-acinar predominant LUAD with an acinar component of ≥5%), N status: nodal status, STAS: tumor spread through air spaces, VPI: visceral pleural invasion, LVI: lymphovascular invasion, WT: wild-type. Note: Bold emphasis is used to indicate statistically significant comparisons. Italic emphasis is used to describe “age” and “tumor size” as a continuous characteristic. Bold emphasis is used to indicate statistically significant *p*-values. The chi-squared test or Fisher’s exact test was used to evaluate the categorical variables, and The Mann–Whitney U test (Wilcoxon Rank Sum Test) was performed to evaluate the continuous characteristics.

**Table 2:** Logistic regression analyses of the *EGFR-Del-19* mutation.

Variable	Univariable		Multivariable	
	*p*-Value	OR (95% CI)	*p*-Value	OR (95% CI)
Acinar: AP (vs. AC)	**0.011**	1.187 (1.101–1.219)	**0.030**	1.951 (1.701–2.205)
TNM stage: II, III (vs. I)	0.791	0.922 (0.490–1.641)		
Age	0.821	1.003 (0.971–1.038)		
Sex: female (vs. Male)	0.146	1.538 (0.877–2.795)		
Smoking: never (vs. ever)	**1.85 × 10^−9^**	7.260 (6.107–8.004)	**1.39 × 10^−7^**	6.22 (5.066–7.060)
Grade: 3 (vs. 1, 2)	**0.007**	0.477 (0.324–0.551)	0.773	0.905 (0.457–1.762)
LVI: presence (vs. absence)	0.330	0.767 (0.446–1.229)		
VPI: presence (vs. absence)	0.231	0.673 (0.337–1.243)		
STAS: presence (vs. absence)	0.058	0.579 (0.331–1.004)		
Lepidic-predominant: Yes (vs. No)	**0.002**	2.198 (1.877–3.022)	0.055	2.158 (1.019–5.021)
Papillary-predominant: Yes (vs. No)	0.665	0.875 (0.695–0.978)		
Micropapillary-predominant: Yes (vs. No)	0.939	0.979 (0.576–1.688)		
Solid-predominant: Yes (vs. No)	**0.019**	0.520 (0.396–0.692)	0.530	0.814 (0.424–1.537)
CGP-predominant: Yes (vs. No)	0.785	0.910 (0.555–1.492)		

AP: acinar-predominant, AC: acinar component (non-acinar predominant LUAD with an acinar component of ≥5%), CGPs: complex glandular patterns (cribriform and fused gland), STAS: tumor spread through air spaces, VPI: visceral pleural invasion, LVI: lymphovascular invasion, OR: odds ratio, CI: confidence interval. Note: Bold emphasis is used to indicate statistically significant *p*-values.

**Table 3:** Univariable and multivariable analysis of RFS using Cox regression hazards model.

Variable	Univariable		Multivariable	
	HR (95% CI)	*p*-Value	HR (95% CI)	*p*-Value
Acinar: AC (vs. AP)	1.358 (1.188–1.541)	**0.006**	1.240 (1.103–1.312)	**0.04**
TNM stage: II, III (vs. I)	2.481 (1.980–3.109)	**2.76 × 10^−15^**	1.830 (1.533–2.118)	**1.25 × 10^−6^**
Age	1.379 (1.265–0.422)	**0.002**	1.293 (1.199–1.321)	**0.014**
Sex: Female (vs. Male)	0.926 (0.738–1.162)	0.506		
Smoking status: Never (vs. Ever)	0.745 (0.427–1.299)	0.300		
Grade: 3 (vs. 1/2)	2.049 (1.816–2.401)	**3.38 × 10^−9^**	1.281 (0.981–1.445)	0.114
LVI: Present (vs. Absent)	1.935 (1.737–2.007)	**2 × 10^−8^**	1.270 (0.974–1.356)	0.076
VPI: Present (vs. Absent)	1.500 (1.391–1.689)	**0.0005**	1.130 (0.985–1.343)	0.324
STAS: Present (vs. Absent)	1.399 (1.279–1.549)	**0.003**	1.119 (0.082–1.220)	0.352
Lepidic-predominant: Yes (vs. No)	0.592 (0.473–0.660)	**4.1 × 10^−6^**	0.859 (0.769–1.003)	0.234
Papillary-predominant: Yes (vs. No)	0.927 (0.721–1.191)	0.555		
Micropapillary-predominant: Yes (vs. No)	1.383 (1.230–1.540)	**0.005**	1.296 (1.107–1.367)	**0.043**
Solid-predominant: Yes (vs. No)	1.551 (1.335–1.646)	**0.0001**	1.008 (0.876–1.127)	0.951
CGP-predominant: Yes (vs. No)	1.417 (1.297–1.513)	**0.005**	1.146 (0.983–1.289)	0.303
KRAS: Mutant (vs. WT)	1.151 (0.921–1.438)	0.214		
EGFR: Mutant (vs. WT)	0.997 (0.715–1.391)	0.989		
BRAF: Mutant (vs. WT)	1.101 (0.655–1.852)	0.716		
MET: Mutant (vs. WT)	0.615 (0.305–1.243)	0.176		

AP: acinar-predominant, AC: acinar component (non-acinar predominant LUAD with an acinar component of ≥5%), CI: confidence interval; HR: hazard ratio, WT: wild-type, LVI: lymphovascular invasion, VPI: visceral pleural invasion, STAS: spread through air space, CGPs: complex glandular patterns (cribriform and fused gland). Note: Bold emphasis is used to indicate statistically significant *p*-values.

## Data Availability

The data presented here are available from the corresponding author Philippe Joubert.

## Supplementary Materials

The following supporting information can be downloaded at: https://www.mdpi.com/article/10.3390/cancers17111825/s1, Table S1: Clinicopathologic and molecular characteristics across LUAD histological patterns.
