# Supplementary material for: A Comprehensive Evaluation of Clinicopathologic Characteristics, Molecular Features and Prognosis in Lung Adenocarcinoma with an Acinar Component
Source: Cancers (Basel). 2025 May 30;17(11):1825. doi: 10.3390/cancers17111825 (PMC12153718; doi:10.3390/cancers17111825)
Supplement: Supplementary file 1 [file cancers-17-01825-s001.zip › cancers-3605050-supplementary.pdf]

Supplementary Table S1. Clinicopathologic and molecular characteristics across LUAD histological patterns.

Characteristics	AP (N=716)	LP (N=136)	PP (N=50)	≥ 20% S/ MP/ CGPs (N=361)	p-value
Age					
Median	*66*	*66*	*67*	*66*	0.528
Min-Max	*37-88*	*45-81*	*50-79*	*26-84*	
**Sex**					
Male	255 (35.6%)	45	30	148	**0.004**
Female	461 (64.4%)	91	20	213	
**Smoking status**					
Ever	675 (94.3%)	10	3	10	**0.0009**
Never	41 (5.7%)	351	47	351	
**N status**					
N0	270 (37.7%)	62	25	140	
N1/2	446 (62.3%)	74	25	221	
**Stage**					
I	543 (75.9%)	134	36	210	**<0.00001**
II	119 (16.6%)	2	7	91	
III	54 (7.5%)	0	7	60	
Tumor size (cm)					
Median	*2.3*	*1.9*	*2.45*	*2.7*	**<0.00001**
Range	*1-15*	*1.0-5.9*	*1.0-12.5*	*1.0-10.5*	
**Type of surgery**					
Lobectomy	539 (75.3%)	89	39	287	**0.0001**
Segmentectomy	75 (10.5%)	30	2	25	
Other	102 (14.3%)	17	9	49	
**Tumor localization**					
Left Upper Lobe	186 (26.0%)	34	9	94	0.065
Left Lower Lobe	84 (11.7%)	11	5	53	
Right Upper Lobe	295 (41.2%)	56	16	119	
Right Lower Lobe	101 (14.1%)	24	12	65	
Other	50 (7.0%)	11	8	30	
**STAS**					
Yes	312 (43.6%)	11	14	200	**<0.00001**
No	404 (56.4%)	125	36	161	
**LVI**					
Yes	329 (46.0%)	9	11	243	**<0.00001**
No	387 (54.0%)	127	39	118	
**VPI**					
Yes	194 (27.1%)	5	11	133	**<0.00001**
No	522 (72.9%)	131	39	228	
**Mutational status**					
KRAS-G12C	149 (20.8%)	21	6	88	**0.024**

KRAS-G12V	67 (9.4%)	16	7	29	0.235
KRAS-G12D	33 (4.6%)	6	3	20	0.858
KRAS-G12A	22 (3.1%)	5	3	9	0.370
KRAS-G12X	12 (1.7%)	2	2	8	0.579
KRAS-G13X	18 (2.5%)	2	3	11	0.249
KRAS-Q61H	19 (2.7%)	3	2	9	0.760
KRAS-Q61L	5 (0.7%)	0	0	6	0.319
EGFR-Del-19	43 (6.0%)	7	2	7	0.122
EGFR-L858R	39 (5.4%)	14	3	6	**0.0001**
EGFR-Ins 20	8 (1.1%)	3	0	2	0.168
EGFR-Other	19 (2.7%)	6	0	10	0.333
MET-Exon 14	23 (3.2%)	13	0	7	**0.0004**
BRAF-V600E	8 (1.1%)	1	0	4	1.000
BRAF-Other	25 (3.5%)	3	1	12	0.919
PIK3CA	7 (1.0%)	2	1	7	1.000
Other	24 (3.4%)	3	3	8	0.251
WT	195 (27.2%)	42	14	125	0.568

AP: acinar predominant, LP: lepidic predominant, PP: papillary predominant, S: solid, MP: micropapillary, CGPs: complex glandular patterns (cribriform and fused gland), N status: nodal status, STAS: tumor spread through air spaces, VPI: visceral pleural invasion, LVI: lymphovascular invasion, WT: wild type.

Note: Bold emphasis is used to indicate statistically significant comparisons.

Italic emphasis is used to describe “age” and “tumor size” as a continuous characteristic.

The chi-squared test or Fisher's exact test, was used to evaluate the categorical variables, and The Kruskal–Wallis was performed to evaluate the continuous characteristics.
